# Impact of integrating guidelines into an antimicrobial stewardship smartphone application on outpatient antibiotic prescribing: a segmented interrupted time series analysis

**DOI:** 10.3389/fdgth.2025.1647528

**Published:** 2025-09-16

**Authors:** Ahmed A. Sadeq, Laila Z. Alhaj Ali, Jinan M. Shamseddine, Barbara R. Conway, Stuart E. Bond, Rizwan Ali, William J. Lattyak, Zahir Osman Eltahir Babiker, Mamoon A. Aldeyab

**Affiliations:** ^1^ Department of Pharmacy, Shaikh Shakhbout Medical City, Abu Dhabi, United Arab Emirates; ^2^ Department of Pharmacy, School of Applied Sciences, University of Huddersfield, Huddersfield, United Kingdom; ^3^ Institute of Skin Integrity and Infection Prevention and Department of Pharmacy, School of Applied Sciences, University of Huddersfield, Huddersfield, United Kingdom; ^4^ Pharmacy Department, Mid Yorkshire Teaching NHS Trust, Wakefield, United Kingdom; ^5^ Statistical Consulting Department, Scientific Computing Associates Corp, River Forest, IL, United States; ^6^ Tropical and Infectious Diseases Division, Sheikh Shakhbout Medical City, Abu Dhabi, United Arab Emirates; ^7^ College of Medicine and Health Sciences, Khalifa University, Abu Dhabi, United Arab Emirates; ^8^ Reading School of Pharmacy, University of Reading, Reading, United Kingdom

**Keywords:** guidelines, antimicrobial stewardship (AMS), smartphone application (App), antibiotic prescribing, interrupted time series analysis

## Abstract

**Introduction:**

Antimicrobial stewardship (AMS) smartphone applications (apps) have been adopted to promote better antimicrobial prescribing practices. We aimed to evaluate the impact of incorporating an app on AMS metrics and adherence to a local antimicrobial guideline in an outpatient setting.

**Methods:**

A quasi-experimental, segmented interrupted time series design was used, involving three study phases (pre-intervention: 1st January 2020 to 31st December 2021; implementation: 1st January 2022 to 31st December 2022, and post-intervention: 1st January 2023 to 30th June 2024) in a hospital outpatient setting. The effect of introducing an AMS app incorporating local antimicrobial guidelines on AMS outcomes was measured.

**Results:**

A total of 24,424 patients were identified. As per the most simple model, the amounts of the following antibiotics, expressed as defined daily dose (DDD) per 100 patient visits, increased significantly during the post-intervention phase: azithromycin (co-efficient 0.297, *p* = 0.007), co-amoxiclav (co-efficient 2.608, *p* = 0.042), and nitrofurantoin (co-efficient 0.908, *p* = 0.003). The trend in fosfomycin use decreased significantly in the post-intervention phase (co-efficient −0.23., *p* < 0.001). Guideline adherence increased significantly after implementing the AMS app (trend change co-efficient 0.011, *p* < 0.001). These changes in antibiotic prescribing represent improved guideline adherence, and are aligned with WHO AWaRe categorisation recommendations.

**Conclusion:**

The app improved the utilization of antibiotic prescribing by increasing adherence to local antimicrobial guidelines, affirming its utility in augmenting AMS in outpatient settings.

## 1 Introduction

The overuse of antibiotics, the rise of drug resistance, and a shortage of new antibiotics in development have combined to create what the Centers for Disease Control and Prevention (CDC) has described as “one of our most serious health threats” (1). Most antibiotic prescriptions are issued in outpatient settings, and the overprescription of these medications is a widespread issue (2, 3). In the Gulf region, including the United Arab Emirates (UAE), the rate of inappropriate antibiotic prescriptions varies by location and can reach as high as 80% (4). A meta-analysis revealed that inappropriate antibiotic prescribing is prevalent throughout the Gulf region, and antimicrobial monitoring initiatives are not consistently implemented in practice (4).

Antimicrobial stewardship (AMS) has become a primary focus to ensure that everyone who needs effective antibiotics, both today and in the future, can access them appropriately (5). AMS was initially implemented in inpatient care, but regulatory and public health organizations also advocate its application in outpatient settings (6). The CDC recommendations for inpatient AMS have been adapted for use in outpatient environments (7). Evidence indicates that AMS initiatives in outpatient settings can optimize antibiotic prescribing without negatively impacting patient outcomes, although effectiveness can vary depending on the type of program implemented (8). Various interventions can be deployed in outpatient settings to enhance antibiotic utilization. One effective method is using clinical decision support systems to assist healthcare professionals (HCPs) make appropriate antibiotic prescribing (9).

AMS initiatives in outpatient settings (6, 7) can effectively rationalize antibiotic prescribing without adversely affecting patient outcomes (8). To successfully apply significant changes in outpatient antibiotic use, outpatient clinicians must have the time and resources necessary to address inappropriate antibiotic prescribing (10). Therefore, the implementation of a clinical decision support system (CDSS), which helps HCPs make appropriate prescribing decisions (9), may enhance antibiotic utilization in such settings (9). With the increasing use of smartphones among HCPs, various AMS smartphone applications (apps) have been developed as CDSS tools, making antibiotic guidelines more accessible in clinical environments (11). Several studies indicate that these apps are widely acceptable in practice, likely due to their ability to provide point-of-care access to important guidelines. However, there is limited evidence to demonstrate that access to these applications significantly influences prescribing behavior or patient outcomes (12–14).

Some studies conducted in inpatient settings have assessed the impact of AMS apps on improving adherence to guidelines and reducing costs (12, 15, 16). However, metrics such as defined daily dose (DDD) and other relevant outcomes have not yet been reported in neither inpatient or outpatient settings. In this study, our primary objective is to evaluate the impact of incorporating an app on AMS metrics and adherence to a local antimicrobial guideline in an outpatient setting and how this is reflected on the DDDs of the prescribed antibiotics. The secondary objective is to check whether the changes are aligned with the WHO AWaRe recommendations.

## 2 Methods

### 2.1 Setting

The study was conducted in the outpatient setting of Shaikh Shakhbout Medical City (SSMC) hospital, a 750-bed tertiary hospital in the Emirate of Abu Dhabi. The hospital provides inpatient and outpatient clinical services as well. It has a well-established AMS program led by a core team of infectious disease physicians, infectious disease clinical pharmacists, microbiologists, and infection prevention and control nurses. The study assessed AMS practices in SSMC's outpatient clinics.

### 2.2 Study design

We used a quasi-experimental, segmented interrupted time series design involving three study phases: pre-intervention (1st January 2020 to 31st December 2021), implementation (1st January 2022 to 31st December 2022), and post-intervention (1st January 2023 to 30th June 2024) to measure the impact of an app on AMS metrics in our outpatient practice. The implementation phase was the period where the app was being implemented.

### 2.3 Inclusion and exclusion criteria

All adult patients aged 18 years and older who were prescribed antibiotics in the outpatient setting were included. Patients younger than 18 and those who had not been prescribed an antibiotic were excluded.

### 2.4 Study intervention

**Figure 1:**
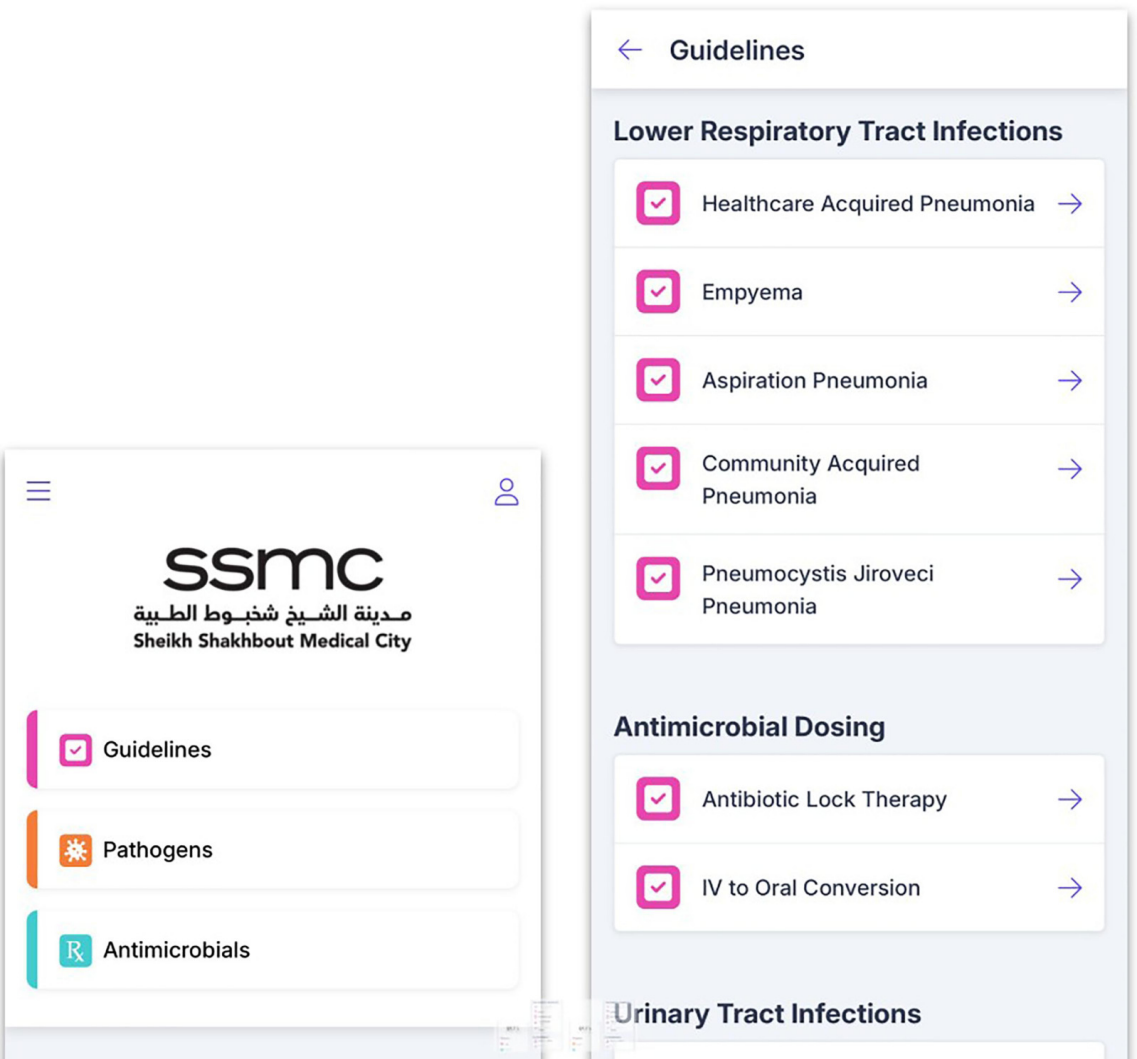
Firstline ™ smartphone application platform. Screenshot from: Firstline app (https://app.firstline.org/).

Previously, SSMC's antimicrobial guidelines were available to inpatient and outpatient settings of the hospital as a document on the hospital's intranet, requiring access through hospital desktops, computers on wheels, or laptops. In this study, an app was introduced to the outpatient settings (Firstline™, https://firstline.org) and the local antimicrobial guidelines were integrated in to it to promote better access and utilization of those guidelines and improve AMS outcomes (17). The hospital's local antimicrobial guidelines were prepared based on the hospital's local antibiogram taking into consideration the WHO AWaRe recommendations. The application provided unrestricted 24/7 antimicrobial guidance and clinical decision support, including bespoke antibiotic choices based on local antibiograms and hospital formulary. The app was made freely available to iOS and Android users, and a web link was provided to replace the previous intranet-hosted guidelines. In order to reach the guidelines for a specific indication, the HCP will choose “antimicrobial guidelines” from the app's main view, then will choose the targeted indication (Figure 1).

### 2.5 Implementation of the intervention

An awareness-raising campaign about the app was launched in 2022 (started in January 2022), and conducted over a period of one year, to increase the app's uptake in the outpatient setting. This campaign included sending emails to staff, distributing physical posters placed around the outpatient clinics, circulating electronic banners to promote the app, and displaying messages on computer screens. Additionally, the AMS team conducted educational sessions for various specialties during departmental meetings. Therefore, the year 2022 (from January 2022 to December 2022) was designated as the implementation phase.

The app's usage statistics were monitored by tracking the monthly numbers of viewing sessions and monthly active users. A user who has used the app at least once during the preceding 28 days was considered a monthly active user. Additionally, utilizing the app for more than 10 s was considered a session. For a fresh session to be logged in after returning, the user would need to exit the app for more than half an hour (17).

### 2.6 Data collection and outcomes

Monthly data were retrospectively collected from the hospital's electronic healthcare system, Cerner™, for the pre-intervention (1st January 2020 to 31st December 2021) and implementation phases (1st January 2022 to 31st December 2022), and were obtained again prospectively for the post-intervention phase (1st January 2023 to 30th June 2024). The following data points were gathered: patient unique medical record number, clinic visit details, age, gender, comorbidities measured by the Charlson Comorbidity Index (CCI) (18), antibiotic prescriptions including type, dosage, and duration, antibiotic consumption recorded in grams, and app utilization data.

In addition, an audit was conducted to measure the monthly levels of adherence to antimicrobial guidelines for each phase. Adherence to antimicrobial guidelines includes adherence to choice and dose. A sample of patients were selected randomly each month from the total population for the auditing purpose. An online calculator (Raosoft, Inc.2004) was used to determine the appropriate sample size that will be selected every month (confidence interval was sat at 95%), and an internet-based randomization system was used to select the patients for the audit (Urbaniak GC, Plous S. Research Randomizer version 4.0) (19). The total selected sample size represented 19% from the total population (3,078/16,453).

The study outcomes included the following measures: antibiotic consumption based on the World Health Organization (WHO) defined daily doses (DDDs) adjusted per 100 patient clinic visits (DDD/100 PV) and categorized based on WHO AWaRe categorization (20), and outpatient antibiotic prescriptions' adherence with local hospital's antimicrobial guidelines. Outcomes were assessed for each calendar month to facilitate time series analysis.

### 2.7 Statistical analysis

Normally distributed continuous data were compared using ANOVA, while nominal data were compared using Cocharan's Q tests. Normally distributed data were expressed as means and standard deviations (SDs), while nominal data were expressed as numbers and percentages.

Interrupted time series analysis was used, applying the Box-Tiao methodology to evaluate whether antibiotic use changed after the app was introduced (21). Because patients' comorbidities may influence antibiotic prescribing, the intervention model used incorporated the mean CCI, which was introduced as a covariate to adjust for variations in patient comorbidities. To compare changes in levels during the implementation and post-intervention phases with the pre-intervention phase, two binary stepwise functions were used: one takes the value “0” before January 2022 and “1” after which captures the immediate level change at the start of the implementation, and the other takes the value “0” before January 2023 and “1” after which captures the additional level shift following full rollout. Additionally, two trend variables were introduced that start at “1” from January 2022 and January 2023, respectively, and increase by “1” thereafter. The implementation trend variable models the gradual effect or momentum during the implementation phase. The second trend variable captures the long-term trajectory or sustained impact after full implementation. Any remaining leftover patterns or correlations among the residuals were addressed using Auto Regressive Moving Average (ARMA) terms in the disturbance model.

The modeling process began by estimating the intervention model as described in the previous paragraph for each antibiotic using an autoregressive model with one lag (AR1) to correct for any serial correlation, which could otherwise bias the results. The residuals (leftover errors) from the model were checked, and the model was adjusted until these residuals looked random and independent. Finally, two versions of the model were produced: one full model with all variables, and a simpler (parsimonious) model where non-significant variables were removed and the statistically significant ones were retained.

All analyses were conducted using SCA Statistical System version 8.1 (Scientific Computing Associates Corp., IL, USA) and SPSS (version 29.0.2.0).

### 2.8 Ethics statement

The study was approved by SSMC's Research Ethics Committee (reference: SSMCREC-381) and the University of Huddersfield Research Ethics Committee, England (reference: SAS-SRIEC-18.12.23-1).

## 3 Results

Table 1 shows the baseline characteristics of the included patients. A total of 24,424 outpatients were included in the study: 9,717 in the pre-intervention phase, 4,727 in the implementation phase, and 9880 in the post-intervention phase. The mean age was 49 years (SD ±18) for the pre-intervention phase, 47 years (SD ±18) for the implementation phase, and 46 years (SD ±18) for the post-intervention phase (*p-value* < 0.001). Males represented 43% (*n* = 4,136) of the pre-intervention phase, 43% (*n* = 2,020) of the implementation phase, and 40% (*n* = 3,926) of the post-intervention phase (*p-value* < 0.001). Additionally, the mean CCI was 1.5 (SD ±2.3) in the pre-intervention phase, 1.8 (SD ±2.2) in the implementation phase, and 1.6 (SD ±2.2) in the post-intervention phase (*p-value* = 0.804).

**Table 1:** Baseline characteristics of outpatients for the pre-intervention (January 2020–December 2021), implementation (January 2022–December 2022), and post-intervention (January 2023–June 2024) phases of the study.

Patients' demographics	Pre-intervention *n* = 9,717	Implementation *n* = 4,727	Post-intervention *n* = 9,880	*P*-Value
Mean Age [years, (SD)]	49 (18)	47 (18)	46 (19)	<0.001
Gender				<0.001
Male	4,136 (43%)	2,020 (43%)	3,926 (40%)	
Female	5,581 (57%)	2,707 (57%)	5,954 (60%)	
Mean CCI (SD)	1.5 (2.3)	1.8 (2.2)	1.6 (2.2)	0.804
Prescribing specialty				
Gastroenterology	2,097 (22%)	988 (21%)	1,611 (18%)	
Urology	1,785 (18%)	620 (13%)	476 (5%)	
Otolaryngology	1,391 (14%)	523 (11%)	1,118 (12%)	
Obstetrics & gynecology	989 (10%)	587 (12%)	1,492 (17%)	
General surgery	813 (8%)	600 (13%)	1,835 (20%)	
Dermatology	580 (6%)	151 (3%)	188 (2%)	
OMF surgery	365 (4%)	247 (5%)	247 (3%)	
Oncology/hematology	313 (3%)	282 (6%)	423 (5%)	
Internal medicine	271 (3%)	269 (6%)	743 (8%)	
Orthopedic	302 (3%)	101 (2%)	116 (1%)	
Pulmonology	252 (3%)	71 (2%)	47 (1%)	
Nephrology	197 (2%)	106 (2%)	170 (2%)	
Ophthalmology	111 (1%)	82 (2%)	213 (2%)	
Rheumatology	97 (1%)	29 (1%)	50 (1%)	
Endocrinology	76 (1%)	32 (1%)	33 (0.004%)	
Cardiology	50 (1%)	24 (1%)	207 (2%)	
Neurology	19 (0.002%)	4 (0.001%)	19 (0.002%)	
Neurosurgery	9 (0.001%)	11 (0.002%)	1 (0.0001%)	
Prescribed antibiotic				
Co-amoxiclav	2,552 (26%)	1,418 (30%)	3,677 (37%)	
Ciprofloxacin	1,369 (14%)	602 (13%)	1,127 (11%)	
Metronidazole	967 (10%)	521 (11%)	814 (8%)	
Amoxicillin	875 (9%)	397 (8%)	733 (7%)	
Levofloxacin	468 (5%)	248 (5%)	527 (5%)	
Nitrofurantoin	454 (5%)	269 (6%)	366 (4%)	
Azithromycin	450 (5%)	179 (4%)	457 (5%)	
Clarithromycin	592 (6%)	217 (5%)	244 (2%)	
Cefuroxime	448 (5%)	228 (5%)	355 (4%)	
Doxycycline	508 (5%)	213 (5%)	305 (3%)	
Clindamycin	366 (4%)	133 (3%)	343 (3%)	
Fosfomycin	153 (2%)	84 (2%)	444 (4%)	
Co-Trimoxazole	123 (1%)	46 (1%)	105 (1%)	
Flucloxacillin	106 (1%)	37 (1%)	119 (1%)	
Moxifloxacin	66 (1%)	45 (1%)	87 (1%)	
Cefixime	59 (1%)	28 (1%)	37 (0.004%)	
Cephalexin	24 (0.002)	17 (0.004%)	54 (1%)	
Cefdinir	82 (1%)	4 (0.0008%)	7 (0.001%)	
Penicillin V	39 (0.004%)	14 (0.003%)	12 (0.001%)	
Linezolid	13 (0.001%)	11 (0.002%)	23 (0.002%)	
Minocycline	3 (0.0003%)	16 (0.003%)	44 (0.004%)	

Continuous data are presented as mean and standard deviation, while nominal data are present as number and percentage.

*n*, number of patients; SD, standard deviation; CCI, Charlson Comorbidity Index; OMF, oral & maxillofacial.

The specialty with the highest number of outpatients who were prescribed antibiotic prescriptions was gastroenterology in the pre-intervention and implementation phases (*n* = 2,097 and *n* = 988, respectively), while general surgery had the highest number in the post-intervention phase (*n* = 1835). The most prescribed antibiotic in all phases was co-amoxiclav (*n* = 2,522, *n* = 1,418, and *n* = 3,677, respectively), and the least prescribed was minocycline in the pre-intervention phase (*n* = 3) and cefdinir in the implementation and post-intervention phases (*n* = 4 and *n* = 7, respectively).

Using segmented analysis, Table 2 presents outpatient antibiotic utilization data according to the WHO AWaRe category, DDD/100 PV, and adherence to antimicrobial guidelines. In addition, Table 3 shows the results of the most simple segmented regression model comparing the involved phases. The CCI was introduced as a covariate in both tables to adjust for potential confounding attributed to patient comorbidity. As per the most simple model, DDD/100 PV of Azithromycin increased significantly in the implementation (level change 0.005, *p* < 0.001) and post-intervention phase (level change 0.297, *p* = 0.007), whereas the DDD/100 PV for co-amoxiclav (level change 2.609, *p* = 0.042) and nitrofurantoin (level change 0.908, *p* = 0.003) increased significantly in the post-intervention phase. The DDD/100 PV level of linezolid (level change 0.045, *p* = 0.006) and trend of levofloxacin (trend change 0.041, *p* = 0.056) increased significantly in the implementation phase. However, the change for both antibiotics became insignificant in the post-intervention phase. The DDD/100 PV trend of cefdinir increased significantly (trend change 0.007, *p* < 0.001) during the pre-intervention phase but that of doxycycline decreased significantly (trend change −0.038, *p* < 0.001). However, the trend change for both antibiotics became insignificant in the implementation and post-intervention phases. The DDD/100 PV trend of fosfomycin decreased significantly during the intervention phase (trend change −0.023, *p* < 0.001). In terms of adherence to the hospital's local antimicrobial guidelines, the level of adherence increased significantly in the post-intervention phase (level change 0.011, *p* < 0.001).

**Table 2:** Segmented interrupted time series analysis of the pre-intervention (January 2020–December 2021), implementation (January 2022–December 2022), and post-intervention (January 2023–June 2024) phases after implementing an antimicrobial stewardship mobile application.

Outcomes	Pre-intervention January 2020–December 2021				Implementation January 2022–December 2022				Post-intervention January 2023–June 2024			
	Intercept	*p*	Trend	*P*	Level change	*p*	Trend change	*p*	Level change	*p*	Trend change	*p*
AWaRe Categorization												
Access%	0.640	0.396	−0.001	0.360	−0.015	0.370	0.007	0.183	0.030	0.305	−0.011	0.071
Watch%	0.3633	0.396	0.001	0.363	0.011	0.384	−0.007	0.206	−0.034	0.283	0.011	0.079
Reserve %	0.000	0.397	0.000	0.355	0.006	<0.001	−0.001	0.019	0.003	0.080	0.001	**0** **.** **017**
DDD/100 PV												
Amoxicillin^†^	5.110	0.133	−0.017	0.380	−0.313	0.380	0.070	0.357	0.384	0.370	−0.084	0.344
Azithromycin^*^^,^^‡^	0.865	0.378	−0.017	0.081	0.227	0.237	−0.008	0.386	0.488	0.059	0.027	0.273
Cefdinir^‡^	0.479	0.394	−0.020	0.000	0.052	0.337	0.019	0.137	−0.016	0.392	0.003	0.386
Cefuroxime^†^	1.724	0.162	0.012	0.385	−0.931	0.249	0.036	0.383	−2.038	0.052	0.150	0.223
Cefixime^‡^	0.142	0.395	−0.003	0.261	−0.052	0.308	0.008	0.296	−0.072	0.266	0.001	0.394
Cephalexin^‡^	0.017	0.396	0.001	0.329	−0.060	0.177	0.005	0.299	0.046	0.271	−0.007	0.228
Ciprofloxacin^‡^	2.017	0.308	−0.034	0.131	0.077	0.393	−0.060	0.278	1.368	**0** **.** **020**	0.039	0.342
Clarithromycin^†^	5.013	0.136	−0.041	0.310	0.152	0.393	−0.106	0.312	1.225	0.202	0.055	0.374
Clindamycin^*^^,^^‡^	1.394	0.324	−0.020	0.237	−0.475	0.228	0.085	0.162	0.422	0.275	−0.092	0.143
Co-amoxiclav^*^^,^^†^	6.747	0.041	−0.083	0.249	−1.027	0.316	0.287	0.175	2.609	0.095	−0.319	0.155
Doxycycline^‡^	3.470	0.242	−0.068	**0** **.** **037**	0.004	0.397	0.056	0.341	0.638	0.283	−0.072	0.305
Flucloxacillin^*^^,^^†^	0.745	0.382	−0.016	0.122	−0.181	0.261	0.056	0.058	−0.050	0.385	−0.040	0.157
Fosfomycin^‡^	0.150	0.394	−0.004	0.223	0.040	0.360	0.003	0.384	−0.045	0.360	0.028	**0** **.** **038**
Levofloxacin^*^^,^^†^	1.753	0.282	0.005	0.393	−0.937	0.109	0.174	0.050	0.456	0.294	−0.237	**0** **.** **013**
Linezolid^†^	0.020	0.396	−0.001	0.303	0.127	**0** **.** **002**	−0.010	0.071	0.095	**0** **.** **042**	0.009	0.106
Metronidazole^‡^	2.641	0.327	−0.013	0.314	−0.395	0.264	0.085	0.155	0.317	0.318	−0.110	0.086
Minocycline^‡^	0.232	0.382	0.000	0.397	−0.065	0.374	0.039	0.143	0.278	0.177	−0.065	0.027
Moxifloxacin^‡^	0.261	0.392	−0.015	0.006	0.152	0.153	0.018	0.199	0.165	0.151	−0.013	0.285
Nitrofurantoin^*^^,^^‡^	1.040	0.343	−0.041	**0** **.** **014**	0.063	0.391	0.001	0.397	1.236	**0** **.** **007**	−0.049	0.259
Penicillin V^*^^,^^‡^	−0.081	0.393	0.022	**0** **.** **000**	−0.404	**0** **.** **000**	−0.003	0.389	0.384	0.370	−0.022	0.116
Total^†^	32.927	<0.001	−0.394	**0** **.** **149**	−3.550	0.303	0.850	0.197	5.655	0.202	−0.794	0.228
Adherence to antimicrobial guidelines^‡^												
	0.164	0.397	0.006	**<0.001**	−0.041	0.081	0.017	**<0.001**	−0.001	0.397	−0.014	**<0.001**

Charlson's comorbidity index was introduced as a covariate.

DDD/100 PV, defined daily dose adjusted over 100 patient visits to the outpatient clinic. Bold values indicate statistically significant results.

* Part of local hospital antimicrobial guidelines.

^†^ Disturbance noise model: (0,1).

^‡^ Disturbance noise model: (0,0).

**Table 3:** Most simple (parsimonious) segmented regression model for the pre-intervention (January 2020–December 2021), implementation (January 2022–December 2022), and post-intervention (from January 2023 to June 2024) phases after implementing an antimicrobial stewardship mobile application.

Outcomes	Pre-interventionJanuary 2020–December 2021				Implementation January 2022–December 2022				Post-intervention January 2023–June 2024			
	Intercept	*p*	Trend	*P*	Level change	*p*	Trend change	*p*	Level change	*p*	Trend change	*p*
DDD/100 PV												
Azithromycin^*^^,^^‡^	0.641	0.396	NA	NA	0.005	0.001	NA	NA	0.297	0.007	NA	NA
Cefdinir^‡^	0.289	0.397	0.007	<0.001	NA	NA	NA	NA	NA	NA	NA	NA
Co-amoxiclav^*^^,^^†^	7.157	0.338	NA	NA	NA	NA	NA	NA	2.609	0.042	NA	NA
Doxycycline^*^^,^^‡^	3.118	0.380	−0.038	<0.001	NA	NA	NA	NA	NA	NA	NA	NA
Fosfomycin^‡^	0.135	0.397	NA	NA	NA	NA	NA	NA	NA	NA	−0.023	<0.001
Levofloxacin^*^^,^^†^	1.482	0.381	NA	NA	NA	NA	0.041	0.056	NA	NA	NA	NA
Linezolid^†^	0.030	0.397	NA	NA	0.045	0.006	NA	NA	NA	NA	NA	NA
Nitrofurantoin^*^^,^^‡^	1.072	0.365	−0.045	<0.001	NA	NA	NA	NA	0.908	0.003	NA	NA
Adherence to antimicrobial guidelines												
	0.173	0.397	NA	NA	NA	NA	0.015	<0.001	NA	NA	0.011	<0.001

Charlson's comorbidity index was introduced as a covariate to adjust for variations in patients’ comorbidities.

DDD/100 PV, defined daily dose adjusted over 100 patient visits to the outpatient clinic; NA, not applicable.

* Part of local hospital antimicrobial guidelines.

^†^ Disturbance noise model: (0,1).

^‡^ Disturbance noise model: (0,0).

.

**Figure 2:**
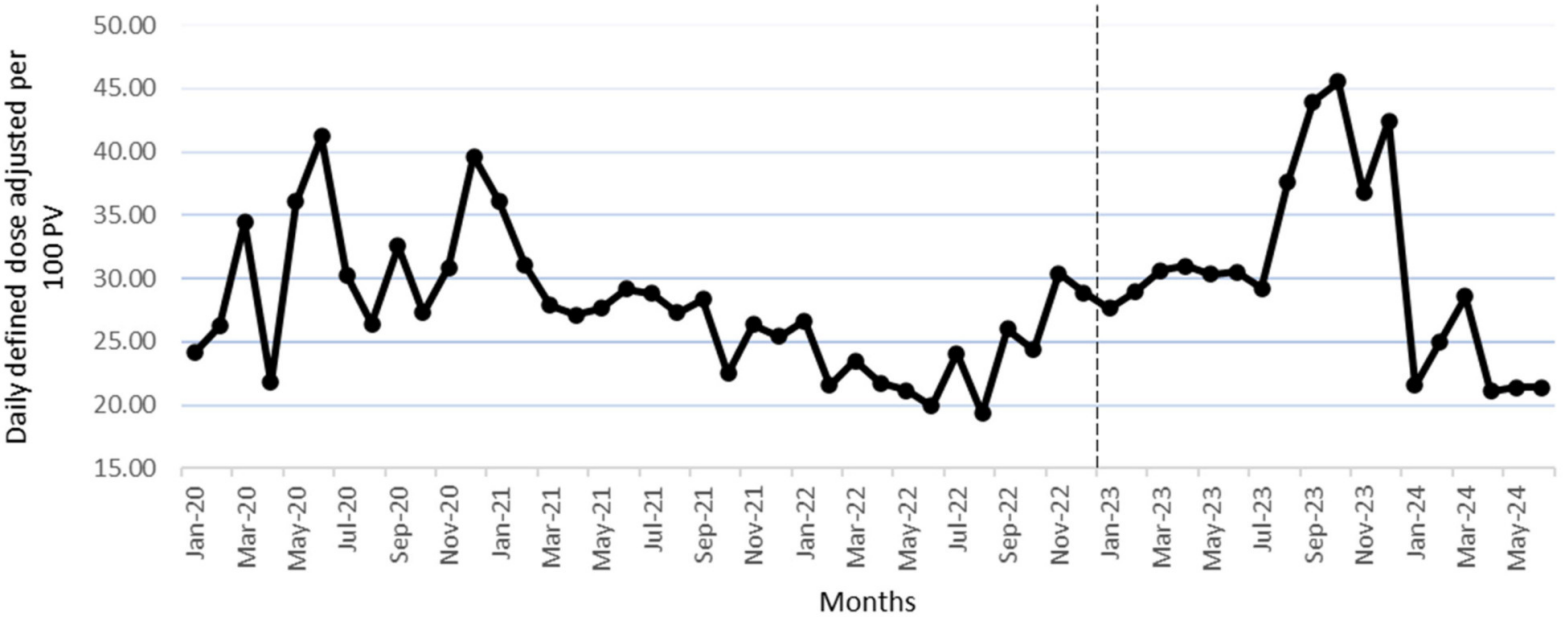
Monthly antibiotic DDD/100 PV from January 2020 to June 2024. The vertical dashed line marks the beginning of the post-intervention phase (January 2023–June 2024).

**Figure 3:**
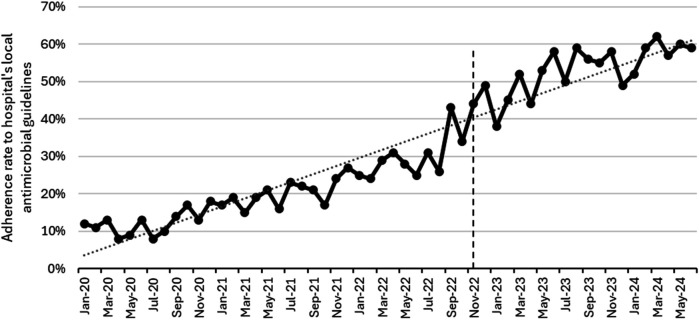
Monthly adherence to hospital antimicrobial guidelines from January 2020 to June 2024. The vertical dashed line represents the start of the post-intervention phase (January 2023–June 2024).

**Figure 4:**
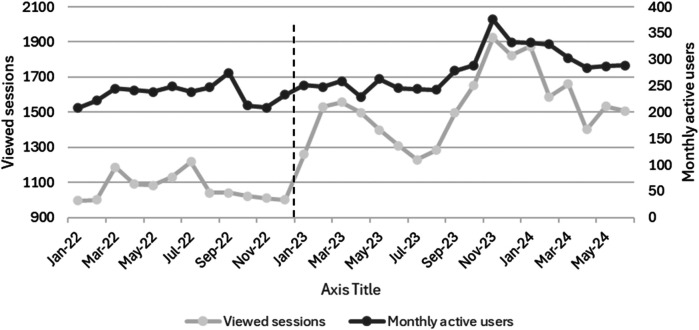
Utilization of the antimicrobial stewardship application during the implementation (January 2022–December 2022) and post-intervention (January 2023–June 2024) phases. The vertical dashed line represents the start of the post-intervention phase (January 2023–June 2024).

Figure 2 shows the monthly DDD/100 PV during the study, while Figure 3 shows the proportion of outpatients that were prescribed antibiotic prescriptions compliant with the hospital guidelines. Furthermore, Figure 4 presents the usage statistics of the AMS app.

## 4 Discussion

This study investigated the impact of integrating local hospital antimicrobial guidelines into an app, branded as “Firstline”, on antibiotic use in the outpatient setting of the study site hospital. The app offers the prescribers access to those guidelines with just three clicks through their smartphones.Three phases were studied: pre-intervention, implementation, and post-intervention. Data of the app utilization were captured to make sure it was used by antibiotic prescribers in the outpatient setting and to monitor the app utilization pattern. The study also provides valuable insights and was associated with improved adherence to the hospital's local antimicrobial guidelines as well as optimized antibiotic use and consumption of antibiotics.

In terms of DDD/100 PV of the antibiotics, the levels of azithromycin, co-amoxiclav, and nitrofurantoin increased significantly in the post-intervention phase, which indicates adherence to the guidelines as these antibiotics are first choice antibiotics for different indications (22). In addition, the increase in nitrofurantoin and co-amoxiclav levels aligns with WHO recommendations, given that those antibiotics are in the “Access category” (23). Furthermore, the use of fosfomycin decreased significantly in the post-intervention phase, which is in line with the hospital antimicrobial guidelines recommendations, in addition to being part of the WHO “Watch category” (23).

The level of linezolid and the trend of levofloxacin increased significantly in the implementation phase; this stopped in the intervention phase as those two antibiotics were not part of the hospital's antimicrobial guidelines, with linezolid being in the “Reserve” while levofloxacin is in the “Watch” WHO AWaRe antibiotic categorization (23), so having non-significant increase in level or trend for those antibiotics is considered a positive outcome. The same applies to cefdinir, as the cessation of its significant trend increase that was there in the pre-intervention phase aligns with the hospital's guidelines and is a desired outcome given that cefdinir is in the “Watch” WHO AWaRe categorization. On the other hand, the doxycycline trend decreased in the non-intervention phase, which then became insignificant in the intervention phase, which is consistent with the hospital's guidelines, making it a desired outcome given that it is in the “Access” WHO AWaRe antibiotic categorization (23). Future efforts should focus on implementing WHO AWaRe recommendations and setting specific targets for the consumption proportions of the three categories: Access, Watch, and Reserve.

This study is the first to investigate the impact of AMS apps on antibiotics' DDD in the outpatient setting. Only one previous study was conducted in Canada, which measured antibiotics' DDD in the inpatient setting and found a significant reduction in inpatient antibiotic utilization represented by DDD/1000 patient days (16). Two studies have evaluated the impact of apps on antibiotic consumption in the inpatient setting, but without using any AMS metrics, and both of them concluded that there was a significant increase in narrow-spectrum antibiotics and a significant reduction in broad-spectrum antibiotics that were recommended by local hospital antimicrobial guidelines (24, 25). These results are consistent with our study findings.

The adherence to the hospital's local antimicrobial guidelines, available on the app, significantly increased during the implementation and post-intervention phases. Simultaneously, we noted a consistent rise in the app's utilization metrics. These findings are consistent with multiple studies that proved the advantage of apps as a clinical decision support system tool to increase adherence to the healthcare facility's local guidelines (26–29). Abdeen and colleagues found out that utilizing an app was linked to greater adherence to a hydration regimen protocol in a study conducted in tertiary academic center in the United States of America (USA) (26). In another study, which was conducted in a primary care clinic in USA, the use of an app encouraged adherence to treatment plans (27). A meta-analysis of thirteen studies concluded that the use of AMS apps appears to boost hospital adherence to guidelines and facilitate access to and awareness of antibiotic prescribing policies (28). In one study in New Zealand, an app was tested among 145 HCPs and found out that the availability of antibiotic guidelines integrated into an app greatly improved provider adherence to such guidelines (29).

This study has some limitations. As the pre-intervention patient phase was retrospectively reviewed, it was not feasible to account for all potential confounding factors that might influence antibiotic utilization. Additionally, since this research was conducted at a single center, additional studies are necessary to gather data that can be generalized to a broader population. Lastly, due to the retrospective nature of the pre-intervention group, the types of infection could not be recalled accurately and were therefore excluded from the analysis.

## 5 Conclusion

We conclude that apps are valuable tools for supporting decision-making to enhance AMS in outpatient settings. They positively affect adherence to guideline which, in turn, affect the AMS metrics related to antibiotic use. However, further research is necessary to assess the impact of these apps on clinical outcomes and cost savings in outpatient care.

## Data Availability

The data that support the findings of this study are available from the corresponding author upon reasonable request and will be subject to the permission of Sheikh Shakhbout Medical City, Abu Dhabi, United Arab Emirates.
